# Pressure injuries as adverse events in the context of patient’s safety during 2013–2020 in a specialist hospital: A retrospective study

**DOI:** 10.1371/journal.pone.0358073

**Published:** 2026-09-30

**Authors:** Izabela Marcelina Sowińska, Sylwia Kocur, Mirosława Noppenberg, Grażyna Puto, Iwona Malinowska-Lipień, Agnieszka Gniadek

**Affiliations:** 1 Department of Internal Medicine and Geriatric Nursing, Institute of Nursing and Midwifery, Faculty of Health Sciences, Jagiellonian University Medical College, Kraków, Poland; 2 Department of Specialized Nursing, Institute of Nursing and Midwifery, Faculty of Health Sciences, Jagiellonian University Medical College, Kraków, Poland; 3 Department of Environmental Nursing, Institute of Nursing and Midwifery, Faculty of Health Sciences, Jagiellonian University Medical College, Kraków, Poland; Universiti Sains Malaysia, MALAYSIA

## Abstract

**Background:**

Hospital-acquired pressure injuries (HAPIs) are recognized as potentially preventable adverse events and important indicators of patient safety and healthcare quality. However, evidence regarding their occurrence within hospital adverse event reporting systems remains limited.

**Objective:**

To assess the occurrence of hospital-acquired pressure injuries recorded as adverse events and to identify factors associated with their occurrence.

**Materials and methods:**

This retrospective study was conducted at a hospital in Kraków, Poland. A total of 1,272 adverse event reports recorded between 2013 and 2020 were reviewed. Among these, 305 hospital-acquired pressure injuries (HAPIs) were identified. A comparison group of hospitalized patients without HAPIs was selected for comparative analyses. Clinical and demographic information was extracted from patients’ medical records.

**Results:**

Between 2013 and 2020, a total of 1,272 adverse events were recorded, representing 1.16% of all hospitalizations during the study period. Hospital-acquired pressure injuries (HAPIs) accounted for 24.0% (305/1,272) of all reported adverse events. Compared with the comparison group, patients with HAPIs had significantly longer hospital stays (18.64 ± 13.51 vs. 12.62 ± 7.81 days; *p* < 0.001) and were prescribed a significantly higher number of medications (18.14 ± 9.03 vs. 10.43 ± 7.90; *p* < 0.001). The highest number of adverse events was reported in the Internal Medicine and Allergology Ward. In multivariable analysis, older age, prolonged hospitalization, a higher number of prescribed medications, and greater comorbidity burden were independently associated with HAPI occurrence.

**Conclusions:**

Hospital-acquired pressure injuries are recognized as an important indicator of patient safety and healthcare quality. Their occurrence was significantly associated with prolonged hospitalization and greater pharmacological burden, suggesting an increased risk among patients with higher clinical complexity. Systematic monitoring of HAPIs within hospital adverse event reporting systems may facilitate the identification of high-risk patients, support targeted preventive interventions, and contribute to improvements in patient safety.

## Background

Pressure injuries remain a significant clinical and organizational challenge in healthcare systems worldwide, despite advances in prevention, standardization of nursing care, and the implementation of risk assessment tools [1]. In recent years, hospital-acquired pressure injuries (HAPIs) have increasingly been classified as potentially preventable adverse events, and their incidence is used as an indicator of quality of care and patient safety. Pressure injuries are viewed not only as a clinical problem but also as the result of complex interactions between patient condition and organizational aspects of care. Previous research has primarily focused on identifying individual risk factors for the development of pressure injuries, whereas relatively few studies have adopted a systemic perspective, incorporating long-term trends, comparisons between patients with and without adverse events, and organizational patterns related to event reporting [2,3]. Studies indicate that the occurrence of pressure injuries is associated with prolonged hospitalization, an increased risk of complications, higher mortality, and a substantial economic burden on healthcare systems [4,5]. The costs of treating hospital-acquired pressure injuries (HAPIs) are estimated to be among the highest of all adverse events recorded in hospitals [4,6]. The development of pressure injuries is influenced by numerous clinical and organizational factors. Patients at particularly high risk include older adults, individuals with multimorbidity, reduced mobility, and cognitive impairment [5,7]. Polypharmacy also plays a significant role, as it is considered an indicator of clinical complexity and a factor increasing susceptibility to adverse events [5,8]. Studies have demonstrated that a higher number of prescribed medications correlates with longer hospital stays, greater levels of dependency, and an increased risk of skin-related complications [5]. Increasing attention has also been given to system-level factors, such as nursing workload, ward profile, and the availability of care resources. Evidence indicates that higher staff workload and personnel shortages are significantly associated with poorer treatment outcomes and a higher incidence of adverse events, including pressure injuries [8]. An especially underexplored aspect is the timing of pressure injury identification, including the time of day and seasonal patterns of reporting, even though these factors may influence the interpretation of registry data and the evaluation of preventive measures [9]. From a patient safety perspective, increasing importance is attributed to adverse event reporting systems, which serve as fundamental tools for monitoring the quality of hospital care [3,10]. The World Health Organization, in its Global Patient Safety Action Plan 2021–2030, emphasizes the need to identify and eliminate preventable harm associated with hospitalization, including events such as pressure injuries [10,11]. In Poland, the issue of pressure injuries as adverse events remains insufficiently described in quantitative and comparative terms, particularly with the use of long-term registry data encompassing both patients with documented adverse events and frequency -matched control group of hospitalized patients without such events. Analyses of this type may provide valuable insights into the scale of the problem, its clinical and organizational determinants, and the potential utility of pressure injuries as indicators of patient safety in hospital practice [12].

The aim of this study was to conduct a retrospective analysis of adverse events recorded in a specialized hospital between 2013 and 2020, with particular emphasis on pressure injuries as a potential indicator of patient safety. The study sought to assess the incidence of pressure injuries, their temporal distribution and distribution across hospital wards, as well as to compare the clinical and organizational characteristics of patients in whom an adverse event was recorded with a frequency-matched control group of hospitalized patients without such events.

## Materials and methods

### Study design and characteristics

A retrospective study was conducted based on the analysis of medical records of patients hospitalized in a specialized hospital between 2013 and 2020. The study had a comparative design and included the analysis of adverse events recorded in Adverse Event Reporting Forms, as well as the medical documentation of patients hospitalized without a documented adverse event (control group).

### Study setting and population

The study was conducted at a hospital in Kraków, Poland. Between 2013 and 2020, a total of 1,272 adverse events were identified through the hospital adverse event reporting system. Only hospital-acquired pressure injuries (HAPIs) recorded as adverse events were eligible for inclusion in the HAPI analysis. Pressure injuries present at hospital admission were not classified as adverse events within the hospital reporting system and were therefore not included in this study. Among these adverse events, 305 hospital-acquired pressure injuries (HAPIs) were included in the comparative analysis. A comparison group of hospitalized patients without HAPIs was selected from the same hospital during the same study period. Clinical and demographic information for both groups was extracted from patients’ medical records.

### Inclusion and exclusion criteria

Medical records of patients hospitalized between 2013 and 2020 with an adverse event documented in the Adverse Event Reporting Form were eligible for review. Records with incomplete documentation that prevented verification of patient characteristics or adverse event details (e.g., time of day, month, event location, or nursing care category) were excluded. Reports related to occupational exposure among healthcare personnel and hospital-acquired infections were also excluded because they were outside the scope of the study. Of the 1481 Adverse Event Reporting Forms initially reviewed, 209 were excluded, leaving 1272 adverse events for analysis. Among these, 305 cases of hospital-acquired pressure injuries (HAPIs) were included in the comparative analysis.

### Variables and definitions

Two analytical approaches were applied in this study. First, all 1272 adverse events were analyzed descriptively to determine the frequency and distribution of hospital-acquired pressure injuries (HAPIs) within the hospital adverse event reporting system. Second, a comparative analysis was conducted to evaluate differences between patients with HAPIs and the comparison group. For the comparative analyses, the dependent variable was the presence of a hospital-acquired pressure injury (yes/no).

### Statistical analysis

Statistical analyses were performed using IBM SPSS Statistics, version 26. Descriptive statistics included the calculation of mean (M), standard deviation (SD), median, interquartile range (IQR), as well as minimum and maximum values. The normality of variable distributions was assessed using the Shapiro–Wilk test. Comparisons between groups were conducted using the Mann–Whitney U test for continuous variables and the chi-square (χ²) test of independence or Fisher’s exact test for categorical variables, depending on whether test assumptions were met. Associations between ordinal variables were evaluated using Spearman’s rank correlation coefficient (ρ). The relationship between the calendar quarter and the time of day of pressure injury identification and documentation or change in its stage was analyzed using the Fisher–Freeman–Halton exact test, with p-values estimated via the Monte Carlo method (10.000 simulations, 99% CI). A *p*-value of < 0.05 was considered statistically significant. To identify factors independently associated with hospital-acquired pressure injuries (HAPIs), an unconditional multivariable logistic regression analysis was performed. The dependent variable was the occurrence of HAPIs (yes/no). Variables considered clinically relevant, including age, sex, body mass index (BMI), length of hospital stay, number of prescribed medications, comorbidity burden, hospital ward, nursing care category, and calendar quarter, were entered into the model. Adjusted odds ratios (aORs) with 95% confidence intervals (95% CIs) were calculated.

### Ethics statement

The study was retrospective and based exclusively on the analysis of existing medical records of patients hospitalized between 2013 and 2020. All data were anonymized prior to analysis, and the researchers had no access to information enabling direct patient identification.

The study protocol was approved by the Bioethics Committee of the Jagiellonian University (opinion no. 1072.6120.42.2022 of 23 February 2022). The study was conducted in accordance with the principles of the Declaration of Helsinki.

## Results

### Adverse events recorded during the study period

Between 2013 and 2020, a total of 1,272 adverse events were identified and included in the descriptive analysis, representing 1.16% of all hospitalizations during the study period. Falls without injury constituted the most frequently reported adverse event (47.5%), followed by hospital-acquired pressure injuries (HAPIs) (24.0%) and falls with injury (16.4%). Overall, 305 HAPIs were identified, corresponding to approximately 0.28% of all hospitalizations during the study period. Less frequent adverse events included patient elopement, theft, mechanical injuries, adverse drug reactions, and several other isolated events. The distribution of adverse events is presented in Table 1.

**Table 1:** Distribution of adverse events recorded between 2013 and 2020 (N = 1272).

Type of adverse event	n	%
Falls without injury	604	47.5
Hospital-acquired pressure injuries (HAPIs)	305	24.0
Falls with injury	209	16.4
Patient elopement	64	5.0
Theft	34	2.7
Mechanical trauma	19	1.5
Adverse drug reaction	9	0.7
Allergic transfusion reaction	8	0.6
Accidental removal of vascular access	7	0.6
Human bite	3	0.2
Drug extravasation	3	0.2
Alcohol intoxication	2	0.2
Exacerbation of psychiatric symptoms	2	0.2
Medical procedure error	1	0.1
Suicide attempt	1	0.1
**Total**	**1272**	**100.0**

### Characteristics of patients included in the comparative analysis

Table 2 in Table 2.

**Table 2:** Sociodemographic characteristics of patients with hospital-acquired pressure injuries (HAPIs) and the comparison group.

Variable	Patients with HAPI	Comparison group	Total	Test statistic	*p-*value	Effect size
**Age**				−4.98	<0.001	0.18
*M*ean (*SD*)	74.57 (14.24)	74.56 (7.70)	74.56 (11.45)			
*M*edian (*IQR*)	78.00 (17.00)	73.00 (12.00)	75.00 (14.00)			
**Sex**				0.00	1.000	−0.01
Female	177 (58.0%)	177 (58.0%)	354 (58.0%)			
Male	128 (42.0%)	128 (42.0%)	256 (42.0%)			
**Place of residence**				0.39	0.534	0.003
Rural	76 (24.9%)	83 (27.2%)	159 (26.1%)			
Urban	229 (74.8%)	222 (72.8%)	450 (73.8%)			
**Education level**				5.55	0.235	0.10
No formal education	2 (0.2%)	2 (0.2%)	4 (0.2%)			
Primary education	16 (5.0%)	19 (4.9%)	35 (5.7%)			
Vocational education	205 (67.2%)	193 (63.3%)	398 (65.2%)			
Secondary education	46 (15.1%)	41 (13.4%)	87 (14.3%)			
Higher education	36 (11.8%)	52(16.7%)	88 (14.4%)			
**Employment status**				22.25	<0.001	0.19
Economically active	5 (1.6%)	30 (9.8%)	35 (5.7%)			
Retired	247 (81.0%)	238 (78.0%)	485 (79.5%)			
Disability pensioner	44 (14.4%)	32 (10.5%)	76 (12.5%)			
Receiving social benefits	8 (2.6%)	4 (1.3%)	12 (2.0%)			
Student	1 (0.3%)	2 (0.2%)	2 (0.4%)			

Abbreviations: SD, standard deviation; IQR, interquartile range.

### Incidence of adverse events

Between 2013 and 2020, a total of 1,272 adverse events were recorded, representing 1.16% of all hospitalizations during the study period. The largest proportion of events was reported in the Internal Medicine and Allergology Ward (37.6%), followed by the Internal Medicine and Gastroenterology Ward (13.6%) and the Neurology Ward (12.7%). The ward-specific distribution of adverse events is presented in Table 3.

**Table 3:** Distribution of adverse events across hospital wards, 2013–2020.

Hospital Ward	Number of events (n)	Percentage (%)
Internal Medicine and Allergology	478	37.58
Internal Medicine and Gastroenterology	173	13.61
Neurology	161	12.66
Internal Medicine, Metabolic Diseases and Geriatrics	132	10.38
Rehabilitation	120	9.42
Cardiology	110	8.64
Rheumatology	64	5.03
Orthopedics	24	1.88
Other	10	0.79
**Total**	**1272**	**100.00**

Hospital-acquired pressure injuries (HAPIs) accounted for 24.0% (305/1.272) of all reported adverse events and were subsequently analyzed in detail.

### Length of hospital stay and number of prescribed medications

Patients with hospital-acquired pressure injuries (HAPIs) had significantly longer hospital stays than those in the comparison group (mean ± SD: 18.64 ± 13.51 vs. 12.62 ± 7.81 days; median [IQR]: 15.00 [16.00] vs. 11.00 [9.00]; Z = −12.50, ***p*** < 0.001). They also received a significantly higher number of prescribed medications (mean ± SD: 18.14 ± 9.03 vs. 10.43 ± 7.90; median [IQR]: 17.00 [11.00] vs. 9.00 [10.00]; Z = −8.28, ***p*** < 0.001). Detailed results are presented in Table 4.

**Table 4:** Comparison of hospital stay duration and prescribed medications between patients with hospital-acquired pressure injuries (HAPIs) and the comparison group.

Variable	Patients with HAPIs	Comparison group	Total	Test statistic	*p-*value	Effect size
**Length of hospital stay**				−12.50	<0.001	0.06
*M* (*SD*)	18.64 (13.51)	12.62 (7.81)	15.63 (11.43)			
*Me* (*IQR*)	15.00 (16.00)	11.00 (9.00)	13.00 (12.00)			
**Number of prescribed medications**				−8.28	<0.001	0.03
*M* (*SD*)	18.14 (9.03)	10.43 (7.90)	16.79 (8.59)			
*Me* (*IQR*)	17.00 (11.00)	9.00 (10.00)	15.00 (10.5)			

### Pressure injury stage

Among patients with hospital-acquired pressure injuries (HAPIs), stage II lesions were the most frequently recorded (46.9%), followed by stage III (38.7%). Stage I lesions accounted for 9.8% of cases, whereas stage IV lesions represented 4.3%. Unstageable lesions were rare (0.3%). The distribution of HAPI stages is shown in Fig 1.

**Fig 1:**
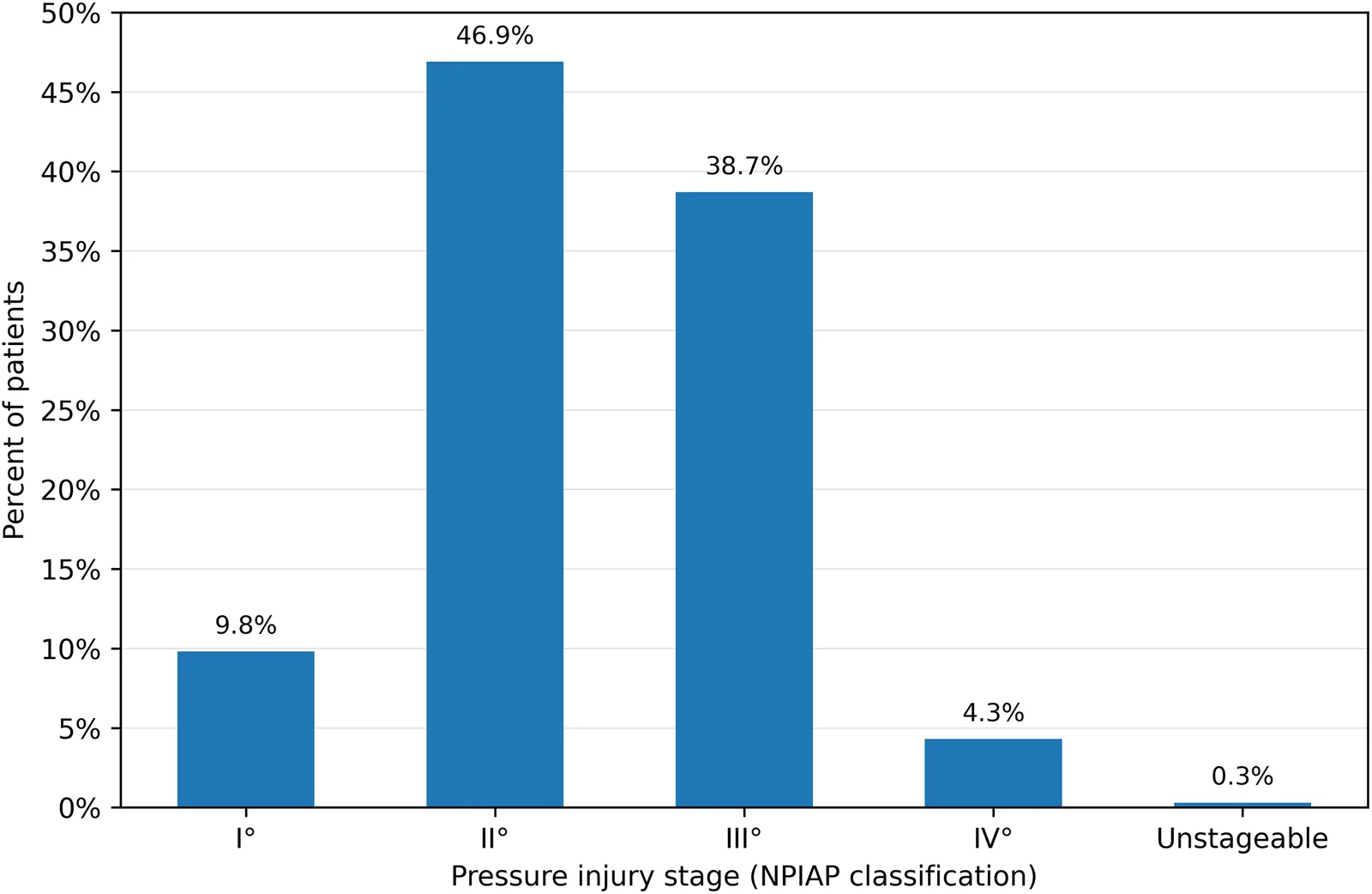
Distribution of hospital-acquired pressure injuries according to pressure injury stage (NPIAP classification).

### Association between medication groups and the total number of prescribed medications

Among patients with hospital-acquired pressure injuries (HAPIs), the total number of prescribed medications differed significantly according to the use of each analyzed medication group (all ***p*** < 0.05). Individuals receiving hypnotics, sedatives, analgesics, diuretics, antidepressants, glucocorticoids, anti-inflammatory drugs, anticoagulants, or antibiotics had a significantly greater pharmacological burden than those who did not receive these medications (Table 5).

**Table 5:** Association between selected medication groups and the total number of prescribed medications among patients with hospital-acquired pressure injuries.

Medication group	Use of medication	Number of prescribed medications		Test statistics		
		Mean rank	*Me* (*IQR*)	*Z*	*p-*value	Effect size
Hypnotics	No (*n* = 170)	135.13	22 (14)	−3.88	<0.001	0.05
	Yes (*n* = 134)	174.54	27 (15)			
Sedatives	No (*n* = 143)	125.00	20 (13)	−5.14	<0.001	0.09
	Yes (*n* = 161)	176.93	27 (15)			
Analgesics	No (*n* = 68)	91.84	18 (11)	−6.46	<0.001	0.14
	Yes (*n* = 236)	169.98	26 (15)			
Diuretics	No (*n* = 46)	85.32	18 (9)	−5.63	<0.001	0.10
	Yes (*n* = 258)	164.48	25 (15)			
Antidepressants	No (*n* = 236)	141.30	22 (12)	−4.14	<0.001	0.06
	Yes (*n* = 68)	191.37	30 (18)			
Glucocorticoids	No (*n* = 161)	131.87	21 (14)	−4.35	<0.001	0.06
	Yes (*n* = 143)	175.73	27 (16)			
Anti-inflammatory drugs	No (*n* = 50)	106.21	19.5 (13)	−4.08	<0.001	0.05
	Yes (*n* = 254)	161.61	25 (15)			
Anticoagulants	No (*n* = 28)	116.98	20 (10)	−2.25	0.025	0.02
	Yes (*n* = 276)	156.10	24 (14)			
Antibiotics	No (*n* = 23)	53.20	15 (8)	−5.64	<0.001	0.10
	Yes (*n* = 281)	160.63	25 (14)			

Abbreviations: n – number of observations; Me – median; IQR – interquartile range; Z – test statistic; p-value – statistical significance.

A significant association was observed between calendar quarter and the time of pressure injury identification (Fisher–Freeman–Halton exact test: χ²(12) = 28.26, *p* = 0.002; Cramér’s V = 0.18). Post hoc analysis with Bonferroni correction showed that morning identifications were more frequent in the first quarter than in the third quarter. In contrast, identifications at noon occurred less frequently in the first quarter than in the second and third quarters (Table 6).

**Table 6:** Relationship between calendar quarter and time of day of pressure injury identification.

Calendar quarter	Q1		Q2		Q3		Q4		χ²(12)	*p-value* [99% *CI*]	*V*_c_	Post hoc comparison of column proportions
Variable	*N*	%	*N*	%	*N*	%	*N*	%				
morning	69	60,5%	29	46,0%	18	32,7%	19	46,3%	28,26	0,002 [<0,001; 0,003]	0,18	I > III
noon	1	0,9%	6	9,5%	5	9,1%	3	7,3%				I < II, III
afternoon	5	4,4%	2	3,2%	6	10,9%	2	4,9%				–
evening	38	33,3%	21	33,3%	22	40,0%	17	41,5%				–
night	1	0,9%	5	7,9%	4	7,3%	0	0,0%				–

Q1 – January–March; Q2 – April–June; Q3 – July–September; Q4 – October–December.

Abbreviations: N – number of observations; χ² – test statistic; p (Monte Carlo) – p-value estimated using the Monte Carlo method; CI – confidence interval; V – Cramér’s V (effect size).

Post hoc comparisons were performed using tests of column proportions with Bonferroni correction for multiple comparisons.

### Independent factors associated with hospital-acquired pressure injuries

Multivariable logistic regression analysis was performed to identify factors independently associated with hospital-acquired pressure injuries (HAPIs). Older age, longer hospital stay, a higher number of prescribed medications, and a greater comorbidity burden were independently associated with increased odds of HAPI occurrence. In contrast, sex was not significantly associated with HAPIs after adjustment for other variables. Detailed results are presented in Table 7.

**Table 7:** Multivariable logistic regression analysis of factors associated with hospital-acquired pressure injuries (HAPIs).

Variable	Adjusted OR (aOR)	95% CI	p-value
Age (per 10 years)	1.41	1.12–1.79	0.004
BMI (per 5 kg/m²)	0.79	0.64–0.98	0.031
Length of hospital stay (per 5 days)	1.52	1.34–1.72	<0.001
Number of prescribed medications (per 5 medications)	1.78	1.54–2.05	<0.001
Number of comorbidities	1.62	1.46–1.79	<0.001
Male sex	1.09	0.68–1.75	0.720

## Discussion

The present study showed that hospital-acquired pressure injuries (HAPIs) accounted for nearly one quarter of all reported adverse events. Compared with the comparison group, patients with HAPIs had significantly longer hospital stays and a greater pharmacological burden. These findings emphasize the considerable clinical and organizational impact of HAPIs and reinforce their importance as indicators of patient safety and healthcare quality. Moreover, patients with longer hospital stays have greater exposure time, increasing the opportunity for both the development and documentation of hospital-acquired pressure injuries. Therefore, prolonged hospitalization should not be interpreted as a direct causal factor. The registration of hospital-acquired pressure injuries constitutes an integral component of adverse event reporting systems and may serve as an indicator of patient safety and hospital care quality. The effectiveness of this process largely depends on the systematic engagement of healthcare personnel in identifying and documenting skin changes, which enables risk monitoring and the planning of preventive interventions. Previous literature emphasizes that the quality of registry data on pressure injuries is closely linked to the maturity of the safety culture within a healthcare institution and the effectiveness of adverse event reporting systems [4,13,14]. The findings of the present study confirm that hospital-acquired pressure injuries remain a significant clinical and organizational challenge. Their occurrence was associated with prolonged length of hospital stay and greater pharmacological burden. Similar observations have been reported in large cohort studies, where the development of a pressure injury was associated with a significant increase in hospital stay, complication rates, and healthcare costs, regardless of ward profile [5,15]. These results are consistent with cohort data indicating that hospital-acquired pressure injuries (HAPIs) significantly contribute to extended hospitalization and increased risk of complications, particularly among older adults and patients with multimorbidity [16]. The weak but statistically significant correlation observed between pressure injury stage and length of hospital stay aligns with previous evidence suggesting that prolonged hospitalization may facilitate the accumulation of risk factors such as reduced mobility, malnutrition, and impaired tissue perfusion [17]. Conversely, the absence of significant differences in pressure injury stage across nursing care categories may indicate that formal patient classification tools do not always fully capture the dynamic nature of risk for advanced skin lesions. This observation supports findings suggesting that standardized assessment tools should be complemented by ongoing clinical evaluation and consideration of ward-specific factors [18]. An important aspect of the present findings is the role of polypharmacy, which was significantly associated with the number of prescribed medications. In contemporary literature, polypharmacy is increasingly viewed not merely as an isolated risk factor but as a marker of clinical complexity. It has been linked to a higher likelihood of adverse events, including cognitive impairment and reduced mobility, which may contribute to the development of pressure injuries [18,19]. The multivariable analysis demonstrated that older age, prolonged hospitalization, greater pharmacological burden, and a higher number of comorbidities remained independently associated with HAPI occurrence after adjustment for potential confounding factors. These findings support the multifactorial nature of pressure injury development and are consistent with previous studies showing that HAPIs occur predominantly among patients with greater clinical complexity.

The temporal differences observed—particularly the higher frequency of pressure injury identification during morning hours and the variation across calendar quarters—likely reflect ward organization, patient assessment routines, and nursing workflows rather than the actual timing of lesion development. Similar patterns have been described in registry-based studies, where event detection was strongly associated with routine care practices (e.g., morning patient assessments) rather than the precise onset of the event [20]. From a patient safety perspective, these findings support the view that hospital-acquired pressure injuries may serve as a meaningful indicator of care quality and preventive effectiveness. According to recent international recommendations, systematic monitoring of HAPIs should form an integral part of comprehensive patient safety improvement strategies, particularly among older patients and those with multimorbidity [21,22]. The implementation of multidimensional prevention programs, incorporating both routine risk assessments and targeted interventions, has been identified as an effective approach to reducing the incidence of HAPIs across diverse clinical settings [9,22].

### Limitations, clinical and organizational implications, and future research directions

The findings of this study should be interpreted in light of several limitations. First, the study had a retrospective design and was based on the analysis of medical records and registry data. Information recorded in the Adverse Event Reporting Forms referred to the time of pressure injury identification by healthcare staff rather than the actual time of lesion development. This may have influenced the observed temporal distribution of events, particularly the higher frequency of registrations during morning hours, which likely reflects ward organization and routine skin assessment procedures rather than the true onset of tissue damage. Second, the study was conducted in a single center, which may limit the direct generalizability of the findings to other healthcare institutions with different organizational structures, staffing patterns, and adverse event reporting practices. Third, because the study was based on routinely collected registry data, some potentially relevant clinical variables were unavailable for analysis. In addition, standardized pressure injury risk assessment scales (e.g., Braden, Norton, or Waterlow) and the anatomical location of pressure injuries were not consistently documented in the available medical records and therefore could not be analysed reliably. Despite these limitations, the study provides important clinical and organizational insights into hospital-acquired pressure injuries recorded as adverse events. The inclusion of a large number of adverse event reports collected over an eight-year period is a major strength of this study and provides a comprehensive overview of hospital-acquired pressure injuries in routine clinical practice.

The observed association between the occurrence and stage of pressure injuries and prolonged hospitalization, as well as greater pharmacological burden, suggests that these events are closely linked to patient clinical complexity and the intensity of therapeutic processes. In this context, pressure injuries may be viewed not solely as nursing-related complications but also as indirect indicators of accumulated risk factors and potential vulnerability in patient safety during hospitalization. From a clinical perspective, the findings underscore the importance of early, systematic, and multidimensional risk assessment for pressure injuries, particularly among patients with extended hospital stays and those exposed to polypharmacy. Polypharmacy, increasingly recognized as a marker of multimorbidity and clinical severity, may affect mobility, cognitive function, and patient cooperation, thereby increasing susceptibility to skin damage. In clinical practice, this highlights the need to consider pharmacological burden as an integral component of comprehensive pressure injury risk assessment rather than merely a numerical variable. The organizational implications primarily relate to the functioning of adverse event reporting systems and the quality of registry data. Recording pressure injuries as adverse events may serve as a valuable tool for monitoring patient safety and hospital care quality. However, its analytical value depends on consistent definitions, standardized timing of skin assessments, and active engagement of healthcare professionals in accurate documentation. The findings suggest that registry-based data on pressure injuries should be interpreted within the broader context of ward organization and clinical routines rather than as a direct reflection of the precise timing of lesion development. Future research should focus on prospective study designs that enable more precise determination of the timing of pressure injury onset and the dynamics of their progression during hospitalization. Further analyses are also warranted to explore the role of polypharmacy in greater detail, including the impact of specific drug classes and changes in treatment regimens during hospital stay. Additionally, multicenter studies would allow comparison of adverse event reporting practices and identification of system-level factors influencing data quality and the effectiveness of preventive strategies. Such approaches may enhance the use of hospital-acquired pressure injuries as meaningful indicators of patient safety and support the development of more effective prevention frameworks.

### Conclusions

Hospital-acquired pressure injuries (HAPIs) remain an important patient safety concern and may serve as valuable indicators of healthcare quality. Their occurrence was significantly associated with prolonged hospitalization and greater pharmacological burden, reflecting increased clinical complexity among affected patients. Continuous monitoring of HAPIs within hospital adverse event reporting systems may support early risk identification, guide preventive interventions, and contribute to ongoing improvements in patient safety and the quality of hospital care. Multivariable analysis confirmed that HAPI occurrence was independently associated with older age, prolonged hospitalization, polypharmacy, and greater comorbidity burden, highlighting the need for targeted preventive strategies in clinically complex patients.

### Study registration

The study did not require registration in a clinical trial registry because of its retrospective, non-interventional design.

## References

[1] Gefen A, Alves P, Ciprandi G, Coyer F, Milne CT, Ousey K, et al. Device-related pressure ulcers: SECURE prevention. J Wound Care. 2020;29(Sup2a):S1–52. doi: 10.12968/jowc.2020.29.sup2a.s1 32067552

[2] Padula WV, Pronovost PJ. Addressing the multisectoral impact of pressure injuries. The Lancet. 2022;399(10338):2029–31. doi: 10.1016/S0140-6736(22)00725-6

[3] Gunningberg L, Hommel A, Bååth C, Idvall E. Pressure injuries as adverse events in healthcare systems. Patient Saf. 2020;16(4):e237–44. doi: 10.1097/PTS.0000000000000509

[4] Padula WV, Delarmente BA. The national cost of hospital-acquired pressure injuries in the United States. Int Wound J. 2019;16(3):634–40. doi: 10.1111/iwj.13071 30693644 PMC7948545

[5] Manzano F, Navarro MJ, Roldán D, Moral MA, Leyva I, Guerrero C, et al. Pressure ulcer incidence and risk factors in ventilated intensive care patients. J Crit Care. 2010;25(3):469–76. doi: 10.1016/j.jcrc.2009.09.002 19879730

[6] Gefen A. The aetiology of medical device-related pressure ulcers and how to prevent them. Br J Nurs. 2021;30(15):S24–30. doi: 10.12968/bjon.2021.30.15.S24 34379465

[7] Mervis JS, Phillips TJ. Pressure ulcers: Pathophysiology, epidemiology, risk factors, and presentation. J Am Acad Dermatol. 2019;81(4):881–90. doi: 10.1016/j.jaad.2018.12.069 30664905

[8] Padula WV, Pronovost PJ. Addressing the multisectoral impact of pressure injuries. Lancet. 2022;399(10338):2029–31. doi: 10.1016/S0140-6736(22)00725-6

[9] Beeckman D, Serraes B, Anrys C, Van Tiggelen H, Van Hecke A, Verhaeghe S. A multicentre prospective randomised controlled clinical trial comparing the effectiveness and cost of a static air mattress and alternating air pressure mattress to prevent pressure ulcers in nursing home residents. Int J Nurs Stud. 2019;97:105–13. doi: 10.1016/j.ijnurstu.2019.05.015 31234104

[10] World Health Organization WHO. Global patient safety action plan 2021–2030: Towards eliminating avoidable harm in health care. Bull World Health Organ. 2021;99(6):429–45. doi: 10.2471/BLT.21.287159

[11] Killaspy H, Baird G, Bromham N, Bennett A, Guideline Committee. Rehabilitation for adults with complex psychosis: Summary of NICE guidance. BMJ. 2021;372:n1. doi: 10.1136/bmj.n1 33436418

[12] Sowińska I, Noppenberg M, Gniadek A. Selected risk factors for adverse events in hospitalised patients in healthcare facilities. Nurs Probl. 2024;32(1):1–6. doi: 10.5114/ppiel.2024.139126

[13] Rochefort CM, Buckeridge DL, Abrahamowicz M. Adverse event reporting systems and patient safety surveillance: A narrative review. BMJ Qual Saf. 2020;29(10):854–63. doi: 10.1136/bmjqs-2019-010241

[14] Slawomirski L, Auraaen A, Klazinga N. The economics of patient safety: strengthening a value-based approach to reducing patient harm. OECD Health Working Papers. 2020. doi: 10.1787/5a9858cd-en

[15] Cox J. Pressure injury risk factors in adult critical care patients: A review of the literature. Ostomy Wound Manage. 2017;63(11):30–43. 29166261

[16] Gethin G, Probst S, Stryja J, Christiansen N, Price P. Evidence for person-centred care in chronic wound care: A systematic review and recommendations for practice. J Wound Care. 2020;29(Sup9b):S1–22. doi: 10.12968/jowc.2020.29.Sup9b.S1 32935648

[17] Jackson D, Durrant L, Bishop E, Walthall H, Betteridge R, Gardner S, et al. Pain associated with pressure injury: A qualitative study of community-based, home-dwelling individuals. J Adv Nurs. 2017;73(12):3061–9. doi: 10.1111/jan.13370 28661013

[18] Maher RL, Hanlon J, Hajjar ER. Clinical consequences of polypharmacy in elderly. Expert Opin Drug Saf. 2014;13(1):57–65. doi: 10.1517/14740338.2013.827660 24073682 PMC3864987

[19] Zullo AR, Gray SL, Holmes HM, Marcum ZA. Screening for medication appropriateness in older adults. Clin Geriatr Med. 2018;34(1):39–54. doi: 10.1016/j.cger.2017.09.003 29129216 PMC9588173

[20] Ding L, Hu X, Wei L, Sun M, Sun G, Jiang G, et al. Risk factors for hospital-acquired and community-acquired pressure injuries: A multicentre mixed case-control study. BMJ Open. 2022;12(4):e057624. doi: 10.1136/bmjopen-2021-057624 35437253 PMC9016407

[21] Edsberg LE, Langemo D, Baharestani MM, Posthauer ME, Goldberg M. Unavoidable pressure injury: State of the science and consensus outcomes. J Wound Ostomy Continence Nurs. 2014;41(4):313–34. doi: 10.1097/WON.0000000000000050 24901936

[22] Huang J, Smith Z, Clarke M. Considerations regarding the hospital-acquired pressure injury admission incidence and severity predictors. J Adv Nurs. 2025;81(5):1123–34. doi: 10.1111/jan.16322

